# Impact of Xpert MTB/RIF for TB Diagnosis in a Primary Care Clinic with High TB and HIV Prevalence in South Africa: A Pragmatic Randomised Trial

**DOI:** 10.1371/journal.pmed.1001760

**Published:** 2014-11-25

**Authors:** Helen S. Cox, Slindile Mbhele, Neisha Mohess, Andrew Whitelaw, Odelia Muller, Widaad Zemanay, Francesca Little, Virginia Azevedo, John Simpson, Catharina C. Boehme, Mark P. Nicol

**Affiliations:** 1 Division of Medical Microbiology and Institute for Infectious Diseases and Molecular Medicine, University of Cape Town, Cape Town, South Africa; 2 Médecins Sans Frontières, Khayelitsha, South Africa; 3 Division of Medical Microbiology, University of Cape Town, Cape Town, South Africa; 4 National Health Laboratory Service, Johannesburg, South Africa; 5 Department of Statistical Science, University of Cape Town, Cape Town, South Africa; 6 Khayelitsha Health, City of Cape Town, Cape Town, South Africa; 7 Foundation for Innovative New Diagnostics, Geneva, Switzerland; University of California San Francisco, United States of America

## Abstract

**Background:**

Xpert MTB/RIF is approved for use in tuberculosis (TB) and rifampicin-resistance diagnosis. However, data are limited on the impact of Xpert under routine conditions in settings with high TB burden.

**Methods and Findings:**

A pragmatic prospective cluster-randomised trial of Xpert for all individuals with presumptive (symptomatic) TB compared to the routine diagnostic algorithm of sputum microscopy and limited use of culture was conducted in a large TB/HIV primary care clinic. The primary outcome was the proportion of bacteriologically confirmed TB cases not initiating TB treatment by 3 mo after presentation. Secondary outcomes included time to TB treatment and mortality. Unblinded randomisation occurred on a weekly basis. Xpert and smear microscopy were performed on site. Analysis was both by intention to treat (ITT) and per protocol.

Between 7 September 2010 and 28 October 2011, 1,985 participants were assigned to the Xpert (*n* = 982) and routine (*n* = 1,003) diagnostic algorithms (ITT analysis); 882 received Xpert and 1,063 routine (per protocol analysis). 13% (32/257) of individuals with bacteriologically confirmed TB (smear, culture, or Xpert) did not initiate treatment by 3 mo after presentation in the Xpert arm, compared to 25% (41/167) in the routine arm (ITT analysis, risk ratio 0.51, 95% CI 0.33–0.77, *p* = 0.0052).

The yield of bacteriologically confirmed TB cases among patients with presumptive TB was 17% (167/1,003) with routine diagnosis and 26% (257/982) with Xpert diagnosis (ITT analysis, risk ratio 1.57, 95% CI 1.32–1.87, *p*<0.001). This difference in diagnosis rates resulted in a higher rate of treatment initiation in the Xpert arm: 23% (229/1,003) and 28% (277/982) in the routine and Xpert arms, respectively (ITT analysis, risk ratio 1.24, 95% CI 1.06–1.44, *p* = 0.013). Time to treatment initiation was improved overall (ITT analysis, hazard ratio 0.76, 95% CI 0.63–0.92, *p* = 0.005) and among HIV-infected participants (ITT analysis, hazard ratio 0.67, 95% CI 0.53–0.85, *p* = 0.001). There was no difference in 6-mo mortality with Xpert versus routine diagnosis. Study limitations included incorrect intervention allocation for a high proportion of participants and that the study was conducted in a single clinic.

**Conclusions:**

These data suggest that in this routine primary care setting, use of Xpert to diagnose TB increased the number of individuals with bacteriologically confirmed TB who were treated by 3 mo and reduced time to treatment initiation, particularly among HIV-infected participants.

**Trial registration:**

Pan African Clinical Trials Registry PACTR201010000255244

*Please see later in the article for the Editors' Summary*

## Abstract 2

Helen Cox and colleagues investigate the impact Xpert MTB/RIF for diagnosing patients with presumptive tuberculosis in a large primary care clinic in Khayelitsha, Cape Town.

*Please see later in the article for the Editors' Summary*

## Editors' Summary

**Background:**

In 2012, about 8.6 million people developed active tuberculosis (TB)—a contagious mycobacterial disease that usually affects the lungs—and at least 1.3 million people died from the disease. Most of these deaths were in low- and middle-income countries, and a fifth were in HIV-positive individuals, who are particularly susceptible to TB. *Mycobacterium tuberculosis*, the bacterium that causes TB, is spread in airborne droplets when people with active disease cough or sneeze. The characteristic symptoms of TB include a cough, weight loss, and night sweats. Diagnostic tests for TB include microscopic examination of sputum (mucus coughed up from the lungs), growth (culture) of *M. tuberculosis* from sputum, and molecular tests (for example, the automated Xpert MTB/RIF test) that rapidly and accurately detect *M. tuberculosis* in sputum and determine its antibiotic resistance. TB can be cured by taking several antibiotics daily for at least six months, although the emergence of multidrug-resistant TB is making the disease harder to treat.

**Why Was This Study Done?:**

To improve TB control, active disease needs to be diagnosed and treated quickly. However, sputum microscopy, the mainstay of TB diagnosis in many high-burden settings, fails to identify up to half of infected people, and mycobacterial culture (the “gold standard” of TB diagnosis) is slow and often unavailable in resource-limited settings. In late 2010, the World Health Organization recommended the routine use of the Xpert MTB/RIF test (Xpert) for TB diagnosis, and several low- and middle-income countries are now scaling up access to Xpert in their national TB control programs. But although Xpert performs well in ideal conditions, little is known about the impact of its implementation in routine (real-life) settings. In this pragmatic cluster-randomized trial, the researchers assess the health impacts of Xpert in a large TB/HIV primary health care clinic in South Africa, an upper-middle-income country that began to scale up access to Xpert for individuals showing symptoms of TB (individuals with presumptive TB) in 2011. A pragmatic trial asks whether an intervention works under real-life conditions; a cluster-randomized trial randomly assigns groups of people to receive alternative interventions and compares outcomes in the differently treated “clusters.”

**What Did the Researchers Do and Find?:**

The researchers assigned everyone with presumptive TB attending a TB/HIV primary health care clinic in Cape Town to receive either Xpert for TB diagnosis or routine sputum microscopy and limited culture. Specifically, Xpert was requested on the routine laboratory request forms for individuals attending the clinic during randomly designated Xpert weeks but not during randomly designated routine testing weeks. During the 51-week trial, 982 individuals were assigned to the Xpert arm, and 1,003 were assigned to the routine testing arm, but because clinic staff sometimes failed to request Xpert during Xpert weeks, only 882 participants in the Xpert arm received the intervention. In an “intention to treat” analysis (an analysis that considers the outcomes of all the participants in a trial whether or not they received their assigned intervention), 13% of bacteriologically confirmed TB cases in the Xpert arm did not initiate TB treatment by three months after enrollment (the trial's primary outcome) compared to 25% in the routine testing arm. The proportion of participants with microbiologically confirmed TB and the proportion initiating TB treatment were higher in the Xpert arm than in the routine testing arm. Finally, the time to treatment initiation was lower in the Xpert arm than in the routine testing arm, particularly among HIV-infected participants.

**What Do These Findings Mean?:**

These findings show that, in this primary health care setting, the provision of Xpert for TB diagnosis in individuals with presumptive TB provided benefits over testing that relied primarily on sputum microscopy. Notably, these benefits were seen even though a substantial proportion of individuals assigned to the Xpert intervention did not actually receive an Xpert test. The pragmatic nature of this trial, which aimed to minimize clinic disruption, and other aspects of the trial design may limit the accuracy and generalizability of these findings. Moreover, further studies are needed to discover whether the use of Xpert in real-life settings reduces the burden of TB illness and death over the long term. Nevertheless, these findings suggest that the implementation of Xpert has the potential to improve the outcomes of TB control programs and may also improve outcomes for individuals.

**Additional Information:**

Please access these websites via the online version of this summary at http://dx.doi.org/10.1371/journal.pmed.1001760.

- The World Health Organization provides information (in several languages) on all aspects of tuberculosis, including general information on tuberculosis diagnostics and specific information on the roll-out of the Xpert MTB/RIF test; further information about the World Health Organization's endorsement of Xpert MTB/RIF is included in a Strategic and Technical Advisory Group for Tuberculosis report; the “Global Tuberculosis Report 2013” provides information about tuberculosis around the world, including in South Africa
- The Stop TB Partnership is working towards tuberculosis elimination and provides patient stories about tuberculosis (in English and Spanish); the Tuberculosis Vaccine Initiative (a not-for-profit organization) also provides personal stories about tuberculosis
- The US Centers for Disease Control and Prevention has information about tuberculosis and its diagnosis (in English and Spanish)
- The US National Institute of Allergy and Infectious Diseases also has detailed information on all aspects of tuberculosis
- The South African National Tuberculosis Management Guidelines 2014 are available
- The Foundation for Innovative New Diagnostics, a not-for-profit organization that helps to develop and introduce new diagnostic tests for tuberculosis, malaria, and neglected tropical diseases, has detailed information about the Xpert MTB/RIF test
- More information about TB, HIV, and drug-resistant TB treatment in Khayelitsha, Cape Town, South Africa are provided by Médecins sans Frontières, South Africa

## Introduction

Despite intensified tuberculosis (TB) control efforts over the last two decades, 8.6 million new TB cases were estimated to have emerged in 2012, resulting in at least 1.3 million deaths [1]. In the absence of dramatic improvements in socio-economic status among individuals at risk for TB, TB control rests on diagnosing prevalent and infectious TB cases, instituting appropriate treatment, and thereby reducing ongoing transmission. Until recently, the mainstay of TB diagnosis in settings with high TB burden was sputum smear microscopy. Smear microscopy, while cheap and easy to implement, has low sensitivity and will therefore detect only TB cases with substantial bacterial load and advanced disease. Sputum culture, while considered the gold standard for pulmonary TB diagnosis, is often available only at the level of a reference laboratory and is most often too slow to be able to guide therapy. This is particularly the case in settings with high HIV prevalence, where the mortality associated with late or inaccurate diagnosis of TB is high [2]. In order to prevent transmission and improve outcomes for individual patients, a diagnostic test that detects TB disease at lower bacterial burdens and provides rapid results in order for treatment to be instituted is needed.

The Xpert MTB/RIF test is a real-time PCR assay for simultaneous detection of *Mycobacterium tuberculosis* and rifampicin resistance [3]. Based on validation and feasibility data [4], the Xpert test was recommended for use in TB diagnosis by the World Health Organization in late 2010. Since then, through preferential pricing for low- and middle-income countries, a number of countries have attempted to scale up access to Xpert MTB/RIF in national TB control programmes [5]. South Africa has committed to provide access to Xpert testing for all individuals with presumptive TB through a staged roll-out strategy nationwide.

While significant data exist on the specificity and sensitivity of Xpert testing [6], there are limited data available on the impact of implementing Xpert on health outcomes in routine programmatic settings [7],[8]. As a result, there remains controversy as to how Xpert should be implemented in different health systems and who should be tested. We aimed to assess the impact of using Xpert for TB diagnosis on yield of TB cases and the timing of TB treatment initiation in a large primary health care clinic, through a pragmatic randomised controlled trial.

## Methods

### Ethics Statement

The study protocol was approved by the University of Cape Town Human Research Ethics Committee (366/2009), and complies with the principles outlined in the Declaration of Helsinki.

### Study Design and Pre-Specified Outcomes

This was a prospective cluster-randomised trial of Xpert implementation compared to the pre-existing routine diagnostic algorithm of smear, culture, and drug susceptibility testing (DST) in a primary care clinic in Khayelitsha, Cape Town, South Africa. This trial, an extension of an earlier validation and feasibility study [4], was registered in the Pan African Clinical Trials Registry (PACTR201010000255244) 1 mo after the study started based on the realisation of the importance of trial registration.

The pre-specified primary outcome was the proportion of bacteriologically confirmed TB cases that had not initiated appropriate treatment by 2 mo after enrolment. For analysis, the denominator for this outcome included all participants diagnosed with bacteriologically confirmed TB (smear, culture, or Xpert) during the 2-mo period after enrolment. As there were a number of TB cases diagnosed late in this period, the time period to assess treatment initiation was extended (at the time of data analysis) to 3 mo after enrolment.

Secondary outcomes were time to diagnosis, time to TB treatment, all-cause mortality, and the number of clinic visits prior to appropriate TB treatment. Two of these secondary outcomes, time to diagnosis and the number of clinic visits prior to treatment, are not reported on here, as data were of poor quality and inconsistently available. Analyses of TB treatment initiation by HIV status, TB treatment outcomes, and yield of rifampicin-resistant TB were conducted post hoc.

### Study Site

The study was conducted in a large primary health care clinic in Khayelitsha, Cape Town. Khayelitsha is a peri-urban township with an estimated population of 400,000 [9]. Antenatal HIV prevalence is estimated at 33%, and TB case notification at 1,200/100,000 [10]. Ubuntu Clinic is a specialised primary care clinic providing integrated HIV and TB services. In 2011, 1,443 individuals received TB treatment at Ubuntu Clinic, and by December 2011, 6,296 clients were receiving antiretroviral HIV treatment through the clinic. In September 2009, a small decentralised laboratory providing sputum smear microscopy, staffed by one laboratory technician, was established on site at Ubuntu Clinic.

### Study Intervention and Randomisation

All adults, aged 18 y and over, presenting at Ubuntu Clinic with presumptive TB were included in the study. The definition of presumptive TB employed in Cape Town includes any of the following: cough of 2 wk or more, weight loss, drenching night sweats, fever of 2 wk or more, chest pain on breathing, or blood-stained sputum. Individuals already receiving anti-TB treatment for 3 d or more were excluded. The study was designed as a pragmatic trial, in order to provide minimal disruption and to reflect normal routine clinical practice. Clinic staff and investigators were unblinded to the intervention. Individual informed consent at study inclusion was waived for this study based on the pragmatic nature of the trial. Randomisation occurred on a weekly basis in order to allow efficient laboratory functioning and use of resources. Each week was randomly allocated as either Xpert or routine diagnostic testing, with the schedule generated prior to the study (using Random.org) by the principal investigator.

**Figure 1:**
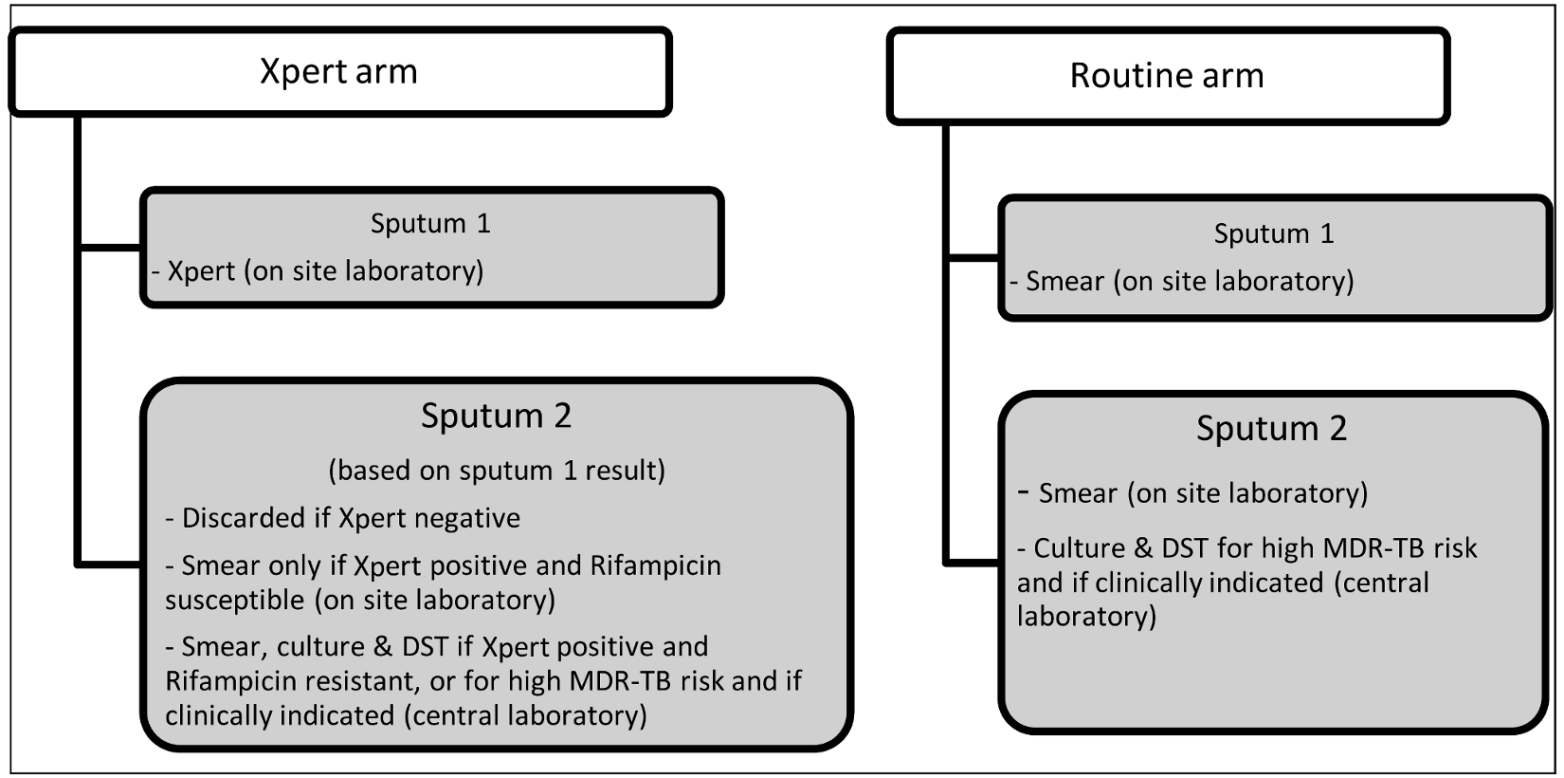
Diagnostic testing algorithm for each study arm.

Patients in both study arms gave two sputum samples as per routine practice in Cape Town. Diagnostic testing for the sputum samples in each arm is described in Figure 1. Diagnosis in the routine arm was based on the policy for diagnostic testing for individuals with presumptive TB in the City of Cape Town. Briefly, diagnosis was primarily based on smear, with culture and DST for those considered at high risk of drug-resistant TB, and where clinically indicated (predominantly among HIV-infected individuals). Individuals considered to be at high risk for drug-resistant TB included those previously treated for TB, health care workers, and those with any prison contact.

Participants enrolled during each designated Xpert week had Xpert requested on the routine laboratory request forms. This was done by trained routine health care staff or one staff member employed specifically for the study and located in the same clinic room in which individuals with presumptive TB were seen. All positive Xpert results were considered as positive for *M. tuberculosis* and requiring treatment. However, at the time of this study, rifampicin resistance diagnosed by Xpert required subsequent confirmation with sputum culture and DST before second-line treatment was initiated.

### Laboratory Methods

Two four-cartridge-capacity GeneXpert machines were installed in the on-site clinic laboratory. The Xpert MTB/RIF test was done on raw sputum samples, with an automated readout provided to the user, as described elsewhere [3]. Indeterminate Xpert results were repeated if further sputum was available. For sputum smear microscopy, in both the on-site and central laboratories, a concentrated auramine smear was prepared and examined under magnification (500×) using a fluorescent microscope and graded according to International Union Against Tuberculosis and Lung Disease guidelines [11]. External quality control for smear microscopy was conducted quarterly. In the central laboratory, a 0.5-ml portion of the sediment was cultured in mycobacterial growth indicator tubes with PANTA and OADC using the BACTEC MGIT 960 system (BD Diagnostics, Sparks, Maryland). Susceptibility to rifampicin was confirmed using the line probe assay MTBDRplus (Hain Lifescience, Nehren, Germany). DST was performed on NaOH-treated pellets for smear-positive sputum and on cultured isolates for smear-negative sputum.

### Participant Follow-Up

Participants were followed up at two time points after study inclusion: at 2–3 mo to assess whether TB treatment was initiated, and at 6 mo to assess mortality. All participants were initially tracked through the clinic TB diagnosis and TB treatment registers to determine status and outcomes. For participants with a negative TB diagnosis and those with a positive diagnosis who were not started on treatment, follow-up consisted of a home visit, with subsequent phone contact if the participant could not be contacted through the home visit. Verbal informed consent was required for collection of follow-up data via telephone contact, and written consent for direct contact with participants.

Additionally, all participants were tracked through district (Cape Town metropole) TB and HIV treatment registers to ascertain whether TB treatment was initiated elsewhere. These registers were also used to ascertain TB treatment outcomes. Participants who could not be followed directly at either of the two follow-up time points were also tracked through regional and national death registries via name and civil identification number, respectively. Linkage with the national death registry in South Africa has been previously shown to have high levels of sensitivity and specificity for Khayelitsha [12].

### Sample Size and Statistical Analysis

The sample size was calculated based on an expected higher yield of bacteriologically confirmed TB cases of 20% to 30% among individuals with presumptive TB in the Xpert arm compared to the routine arm (80% power and one-sided significance *p*<0.05). We assumed 40 patients would be seen per week and assumed a weak intra-cluster correlation coefficient of 0.05, resulting in a design effect of 3 and a required sample size of 882 per study arm.

Bacteriological confirmation of TB was defined as a positive Xpert result for *M. tuberculosis*, *M. tuberculosis* isolated on liquid culture, or positive smear microscopy regardless of grade. Mortality was assessed up to 6 mo after study inclusion. TB treatment outcomes were defined using World Health Organization guidelines [13], with treatment success defined as either cure or treatment completion in the absence of treatment failure. Mortality was defined as death regardless of cause.

The proportion of participants initiating treatment and time to TB treatment initiation were calculated from the date of participant enrolment, with data censored at death. As ascertainment of TB treatment initiation or not by 3 mo was assumed to be complete, data were not censored for loss to follow-up in Kaplan Meier analyses. Similarly, ascertainment of death was assumed to be complete at 6 mo after enrolment.

Analyses were based on intention to treat (ITT), regardless of which diagnostic tests were performed, and per protocol, based on which tests were actually conducted. Response rates and category membership were compared using design-based F-tests to take into account the cluster sampling. Kaplan Meier analyses were used to assess the proportion of participants initiating TB treatment over time, with comparisons using the logrank test.

## Results

### Study Population and Randomisation

In total, 1,985 individuals with presumptive TB were included in the study over the 60 wk from September 7, 2010, to October 28, 2011. The study was interrupted for a total of 9 wk during the study period because of technical or safety issues in the on-site laboratory and sick leave of laboratory staff. During these periods, no participants were recruited, and routine practice was followed.

**Figure 2:**
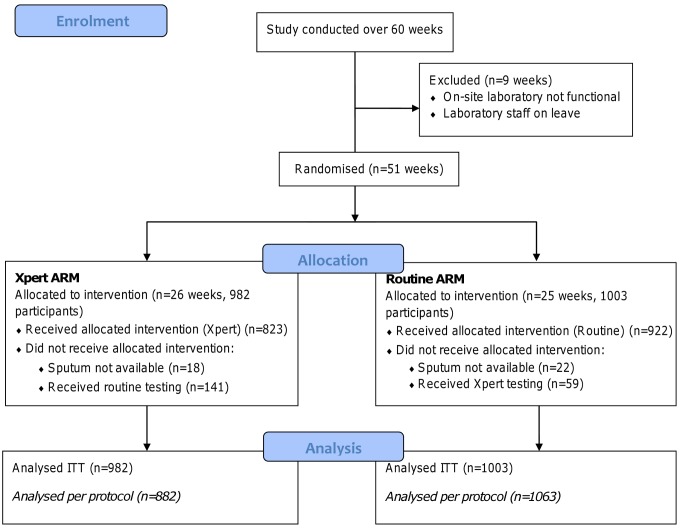
Participant flow diagram.

During the study weeks, the intervention was not always correctly assigned. During 23 of the 51 wk of the study, more than 10% of participants in that week received the incorrect intervention, most commonly routine testing instead of Xpert (participant flow diagram, Figure 2). A review of data and processes suggests that a number of issues contributed to this. The most common cause of incorrect intervention assignment was a failure to request Xpert testing during Xpert weeks, generally resulting from the absence of the specific study health care worker for short periods of time in the clinic consulting room, whereby clinic staff requested sputum tests as per routine. This occurred despite extensive training, and was unfortunately not detected until near the study end. In addition, at the start of some weeks, both routine and study staff continued the intervention that was in place the previous week and this was not corrected until sometime during the week.

The ITT analysis included all 1,985 participants (982 in the Xpert arm and 1,003 in the routine arm), while the per protocol analysis, based on whether participants received Xpert or not, excluded participants where no sputum sample was available, yielding 1,945 participants in total (882 in the Xpert arm and 1,063 in the routine arm). There were no differences in the proportions of individuals with previous TB treatment, known HIV status, and HIV positivity between arms in both analyses, and arms were similar with regard to age and sex (Table 1), suggesting that those participants who did not receive the correct intervention were not different than those who did.

**Table 1:** Clinical and demographic characteristics of participants by study arm for both the ITT and per protocol analyses.

Characteristic	ITT Analysis		Per Protocol Analysis	
	Xpert	Routine	Xpert	Routine
***N***	982	1,003	882	1,063
**Male**	543 (55.3%)	542 (54.0%)	486 (55.1%)	584 (54.9%)
**Age**				
18–30 y	228 (23.2%)	266 (26.5%)	216 (24.5%)	264 (24.8%)
31–40 y	343 (34.9%)	310 (30.9%)	305 (34.6%)	340 (32.0%)
41–50 y	208 (21.2%)	221 (22.0%)	180 (20.4%)	242 (22.8%)
51+ y	203 (20.7%)	205 (20.5%)	181 (20.5%)	316 (20.3%)
**HIV status known**	815 (83.0%)	810 (80.8%)	723 (82.0%)	975 (82.3%)
**HIV positive (percent of known)**	481 (59.0%)	484 (59.8%)	429 (59.3%)	525 (60.0%)
**HIV negative**	334	326	294	350
**Previous TB treatment**	382 (38.9%)	363 (36.2%)	327 (37.1%)	409 (38.5%)

Data are *n* (percent).

### TB Diagnostic Results

In the ITT analysis, 98% (964/982) of participants in the Xpert arm and 98% (981/1,003) of participants in the routine arm received any diagnostic TB testing on sputum (Table 2). The remaining participants either failed to submit any sputum samples, or samples could not be tested, most commonly because of leakage or inadequate specimen labelling.

**Table 2:** Microbiological results by study arm for both the ITT and per protocol analyses.

Result	ITT Analysis			Per Protocol Analysis		
	Xpert	Routine	*p*-Value	Xpert	Routine	*p*-Value
***N***	982	1,003		882	1,063	
**Sputum available**	964 (98.2%)	981 (97.8%)	0.732	882	1,063	
**Xpert**						
Xpert available	823 (85.4%)	59 (6.0%)		882		
Xpert negative	619	47		666		
Xpert indeterminate	5	0		5		
Xpert positive (percent of Xpert available)	199 (24.2%)	12 (20.3%)	0.469	211 (23.9%)		
**Sputum smear microscopy**						
Smear available	676 (68.8%)	958 (95.5%)		571 (64.7%)	1,062 (99.9%)	
Smear negative	593	867		508	951	
Smear positive (percent of smear available)	83 (12.3%)	91 (9.5%)	0.076	63 (11.0%)	111 (10.5%)	0.713
**Culture**						
Culture available	474 (48.3%)	459 (45.8%)	0.476	418 (47.4%)	515 (48.5%)	0.493
Contaminated/lost	29 (6.1%)	29 (6.3%)		29 (6.8%)	29 (5.6%)	
Non-tuberculous mycobacteria	8	2		7	3	
Culture negative	318	319		281	356	
Culture positive (percent of valid^a^ culture results)	119 (27.2%)	109 (25.5%)	0.533	101(26.4%)	127 (26.3%)	0.963
**Bacteriologically confirmed TB (Xpert, smear, or culture)**	257 (26.2%)	167 (16.7%)	<0.001	235 (26.6%)	189 (17.8%)	<0.001

Data are *n* (percent).

a Excludes cultures that are contaminated.

Across both analyses, the proportion of patients whose specimens underwent culture in both arms was similar, as was the proportion of these with positive culture (Table 2). The most common indication for culture was previous TB treatment, with very few participants classified as being at high risk for multidrug-resistant TB (MDR-TB) for other reasons (Table 3). The remaining participants for whom culture was requested were primarily HIV-infected participants with a negative sputum smear (ITT analysis; Table 3). In the ITT analysis, 24% (199/823) of all Xpert tests were positive, while only 10% (91/958) of participants in the routine arm were sputum-smear-positive (predominantly sputum smears conducted in the on-site laboratory). Similar percentages were seen in the per protocol analysis (Table 2). There was a significantly higher rate of bacteriologically confirmed TB disease (by either sputum smear, culture, or Xpert) in the Xpert arm than in the routine arm: 17% (167/1,003) in the routine arm and 26% (257/982) in the Xpert arm (ITT analysis, risk ratio 1.57, 95% CI 1.32–1.87, *p*<0.001), with 18% (189/1,063) and 27% (235/882), respectively, in the per protocol analysis (risk ratio 1.50, 95% CI 1.27–1.78, *p*<0.001) (Table 2).

**Table 3:** Number and percentage of participants for whom culture was requested by indication across study arms (ITT analysis).

Indication for Culture	Xpert		Routine		*p*-Value
	*N*	Culture Requested	*N*	Culture Requested	
Previous TB treatment	382	350 (92%)	363	329 (91%)	
Other MDR-TB risk factors	3	3 (100%)	0	0 (0%)	
HIV-infected, smear-negative (excluding previous TB treatment and other MDR-TB risk factors)	143	72 (50%)	248	84 (34%)	0.438
Reason for culture not stated		49		46	
Total culture requested		474		459	

Among the 211 participants with a positive Xpert result, smear results were available for 95.7% (202/211), but only 28.7% (58/202) of these were sputum-smear-positive. Culture was performed for 96 participants with positive Xpert results, among whom 82 (85.4%) were culture-positive, three had contaminated culture, and 11 were culture-negative. Among individuals with positive Xpert results, there was no difference in the proportion that were culture-negative between those who reported previous TB treatment and those with no previous TB treatment (risk ratio 1.01, 95% CI 0.32–3.2, *p* = 0.99).

### Primary Outcome

Among patients with bacteriologically confirmed disease (smear, culture, or Xpert), 13% (32/257) in the Xpert arm had not initiated treatment by 3 mo after enrolment, compared to 25% (41/167) in the routine arm (ITT analysis, risk ratio 0.51, 95% CI 0.33–0.77, *p* = 0.0052).

### TB Treatment Initiation and Time to Treatment

In the Xpert arm, TB treatment was initiated within 3 mo for a significantly greater proportion of participants, regardless of bacteriological confirmation, in both the ITT and per protocol analyses (Table 4). In the ITT analysis, 28% (277/982) of participants in the Xpert arm started treatment, compared to 23% (229/1,003) in the routine arm (risk ratio 1.24, 95% CI 1.06–1.44, *p* = 0.013). This increased treatment initiation was due primarily to increased treatment initiation among HIV-infected participants, with no significant difference among HIV-negative participants (Table 4). In the Xpert arm, the majority of participants starting treatment had bacteriologically confirmed TB, and the proportion of patients initiating TB treatment without bacteriological confirmation of pulmonary TB, was approximately halved overall (ITT analysis; Table 5).

**Table 4:** TB treatment initiation, overall and by HIV status, in the Xpert and routine arms for both the ITT and per protocol analyses.

Treatment Initiation	ITT Analysis			Per Protocol Analysis		
	Xpert	Routine	*p*-Value	Xpert	Routine	*p*-Value
**All study participants, ** ***n***	982	1,003		882	1,063	
Start TB treatment within 3 mo, *n* (percent)	277 (28.2%)	229 (22.8%)	0.013	256 (29.0%)	246 (23.1%)	0.0052
Days to start treatment^a^, median (IQR)	4 (2–8)	8 (2–27)		4 (2–7)	9 (2–27)	
**HIV-negative participants, ** ***n***	334	326		294	350	
Start TB treatment within 3 mo, *n* (percent)	68 (20.4%)	60 (18.4%)	0.536	60 (20.4%)	66 (18.9%)	0.625
Days to start treatment^a^, median (IQR)	4 (2–7)	11 (3–35)		3 (1.5–6)	13 (4–35)	
**HIV-infected participants (** ***n*** **)**	481	484		429	825	
Start TB treatment within 3 mo, *n* (percent)	171 (35.6%)	127 (26.2%)	0.003	161 (37.5%)	135 (25.7%)	<0.001
Days to start treatment^a^, median (IQR)	4 (2–10)	8 (2–22)		4 (2–7)	8 (2–27)	

a The number of days from enrolment to the start of treatment.

IQR, interquartile range.

**Table 5:** Participants initiating TB treatment with confirmed TB (bacteriologically positive) and unconfirmed TB, overall and by HIV status (ITT analysis).

Participants	Started TB Treatment		*p*-Value
	Xpert	Routine	
**All study participants**	982	1,003	
Confirmed TB	226 (23.0%)	131 (13.1%)	
Unconfirmed TB	51 (5.2%)	98 (9.8%)	0.0025
**HIV-negative participants**	334	326	
Confirmed TB	52 (15.9%)	33 (10.1%)	
Unconfirmed TB	15 (4.5%)	27 (8.3%)	0.1608
**HIV-infected participants**	481	484	
Confirmed TB	139 (28.9%)	68 (14.0%)	
Unconfirmed TB	32 (6.7%)	59 (12.2%)	0.0231

Data are the number (percent) of participants who started TB treatment within 3 mo of enrolment.

**Figure 3:**
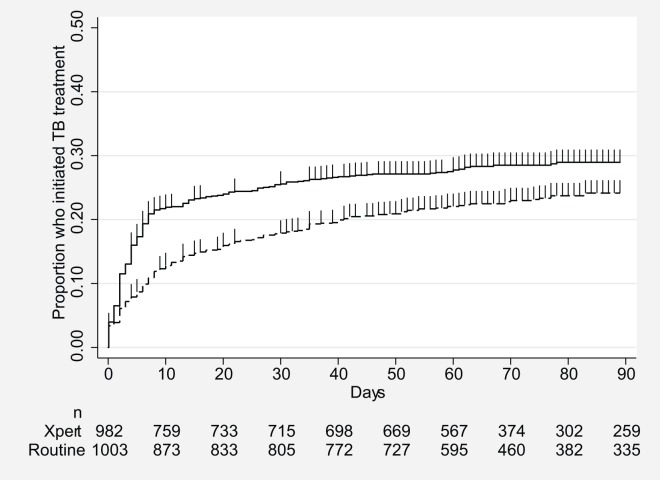
Proportion of participants initiating TB treatment by time to TB treatment initiation for both study arms, ITT analysis (*p* = 0.0042). Xpert: solid line; routine: dashed line.

**Figure 4:**
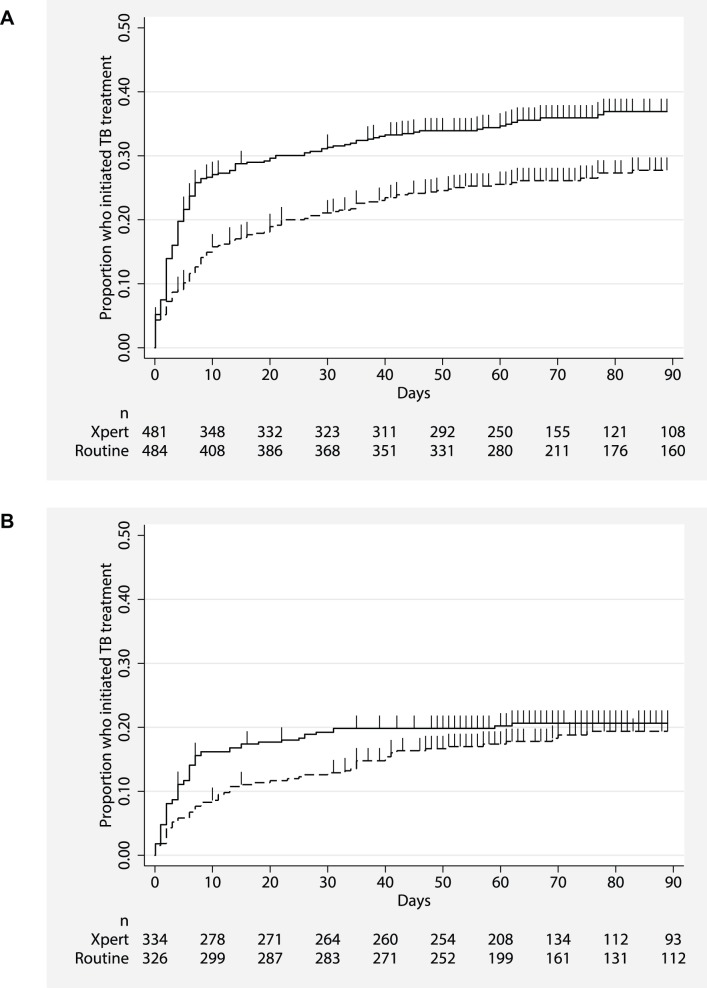
Proportion of HIV-infected and -uninfected participants initiating TB treatment by time to treatment initiation for both study arms, ITT analysis. Xpert: solid line; routine: dashed line. (A) HIV-infected individuals (*p*<0.001); (B) HIV-uninfected individuals (*p* = 0.778).

In addition to increased numbers starting treatment, TB treatment was also initiated more rapidly in the Xpert arm: a median of 4 d after enrolment in the Xpert arm in both analyses compared to 8 and 9 d in the routine arm in the ITT and per protocol analyses, respectively (Table 4). In contrast to the results by HIV status for the proportion starting treatment, reduced delay was more notable among HIV-negative participants than among HIV-infected participants (Table 4). These dual benefits of increased treatment initiation and reduced delay are shown in a time-to-treatment analysis (Kaplan Meier) for all participants (Figure 3) and by HIV status (Figure 4). Time to treatment initiation (Kaplan Meier) was significantly improved overall with Xpert compared to routine testing (ITT analysis, hazard ratio 0.76, 95% CI 0.63–0.92, *p* = 0.005) and among HIV-infected participants (ITT analysis, hazard ratio 0.67, 95% CI 0.53–0.85, *p* = 0.001) (Figures 3 and 4A). Although TB treatment was initiated more rapidly among HIV-negative participants, the proportion starting treatment by 3 mo was similar between Xpert and routine arms (ITT analysis, hazard ratio 0.86, 95% CI 0.60–1.22, *p* = 0.39) (Figure 4B).

### Rifampicin Resistance

There was no difference in the proportion of confirmed rifampicin-resistant TB diagnosed in the two arms in the ITT analysis: 1.1% (11/982) in the Xpert arm and 0.8% (8/1,003) in the routine arm (risk ratio 1.40, 95% CI 0.57–3.48, *p* = 0.4219). Among the 424 bacteriologically confirmed TB cases across both arms, 19 (4.5%, 95% CI 2.8%–6.8%) were confirmed to be rifampicin-resistant.

Of the eight cases found to be Xpert-positive and rifampicin-resistant in the Xpert arm, only five of these were confirmed on culture and line probe assay. Of the remaining three cases, two were deemed rifampicin-susceptible using the line probe assay, and no culture could be obtained for the remaining case. A further two Xpert-negative cases in the Xpert arm were found to be rifampicin-resistant after a positive culture, while two cases were tested as Xpert-positive with rifampicin susceptibility, but were diagnosed as rifampicin-resistant on culture and line probe assay.

### TB Treatment Outcomes and Survival

Among participants initiating treatment (excluding those diagnosed with confirmed rifampicin-resistant TB), TB treatment outcomes were similar, both overall and among HIV-infected participants in the ITT analysis (Table 6). However, given the difference in the proportion of participants initiating treatment, the proportion of participants with a successful treatment outcome overall was significantly greater in the Xpert arm than in the routine arm: 21.9% (215/982) compared to 17.5% (176/1,003), respectively (risk ratio 1.25, 95% CI 1.04–1.49, *p* = 0.0319). This effect was greater for HIV-infected participants: 26.8% (129/481) of participants in the Xpert arm were successfully treated, compared to 19.6% (95/484) in the routine arm (risk ratio 1.37, 95% CI 1.08–1.73, *p* = 0.0119).

**Table 6:** Treatment outcomes for all participants and HIV-infected participants (excluding rifampicin-resistant TB) by study arm (ITT analysis).

Outcome	Xpert	Routine	*p*-Value
**All participants who started TB treatment**	268	224	
Treatment success	215 (80.2%)	176 (78.6%)	0.750
Unfavourable outcome (default, death, failure)	34 (12.7%)	28 (12.5%)	
Transferred or not evaluated	19 (7.1%)	20 (8.9%)	
**HIV-infected participants who started TB treatment**	166	124	
Treatment success	129 (77.8%)	95 (76.6%)	0.492
Unfavourable outcome (default, death, failure)	24 (14.5%)	15 (12.1%)	
Transferred or not evaluated	13 (7.8%)	14 (11.3%)	

Data are *n* (percent).

Within 6 mo from presentation, 3.4% (33/982) of participants died in the Xpert arm and 3.8% (38/1,003) in the routine arm (ITT analysis, risk ratio 0.89, 95% CI 0.56–1.40, *p* = 0.5178). Among HIV-infected participants, 6-mo mortality was similar: 5.0% (24/481) in the Xpert arm and 5.0% (24/484) in the routine arm (risk ratio 1.01, 95% CI 0.58–1.75, *p* = 0.9789).

## Discussion

In this primary care setting with high TB and HIV prevalence, this study demonstrates clear benefits from the provision of Xpert MTB/RIF testing for all individuals with presumptive TB over a diagnostic algorithm relying primarily on smear microscopy. The proportion of individuals with bacteriologically confirmed TB who had not initiated TB treatment by 3 mo, the primary outcome, was significantly lower in the Xpert arm than in the routine arm. In addition, the proportion of participants with microbiologically confirmed TB and the proportion initiating TB treatment were both significantly higher in the Xpert arm. These gains were combined with shortened times to treatment, leading to significant improvements in overall TB treatment initiation and the proportion of participants successfully treated for TB.

These benefits were seen despite inadequate application of the intervention. This was a pragmatic trial aiming to provide minimal disruption and change to normal clinic practices. However, the pragmatic nature of the trial resulted in compromised trial conduct. Based on a retrospective review of practices, we do not feel that the inadequate application of the intervention was due to any systematic biases by health care staff. There was no evidence that Xpert was more likely to be requested for patients who might be considered to be at higher risk of TB or MDR-TB. As there was no significant difference between the Xpert arm and the routine arm in the proportion of smear-negative HIV-infected participants for whom culture was requested, this is not expected to have contributed to the higher rate of bacteriologically confirmed TB in the Xpert arm, nor to the proportion of participants initiating TB treatment rapidly after presentation. Overall, we feel that the problems with application of the intervention were predominantly because of the crossing over of the intervention using the weekly randomisation schedule and the difficulties of trying to introduce additional steps into a well-established clinical process.

Similarly, the intervention was unblinded, and this is also a limitation of the study design chosen. The study also included only participants who gave sputum samples and were diagnosed with TB at the study clinic. Considerably more patients than these were registered for TB treatment at the clinic. The majority of these additional patients were diagnosed elsewhere but were transferred to the study clinic to receive treatment. A small group of patients were started on TB treatment without attempting to produce a sputum sample, against the diagnostic algorithm in place at the clinic. These patients were therefore not included in the study.

Conduct of the study in a single, well-researched clinic may also be considered a limitation. However, differences in the patient populations served by different clinics suggest that a large number of clinics would be required if the study were to be conducted across multiple clinics, and, for this study, it was not logistically or economically feasible to install on-site laboratories in multiple smaller clinics. Comparisons before and after the introduction of new diagnostic algorithms are also subject to confounding by concurrent changes in practices. Overall, although there were several limitations with the pragmatic approach chosen in this study, we believe that the results obtained represent the closest to those that would be seen under programmatic conditions, while balancing a comparison with a valid control group. More interventionist approaches are unlikely to reflect routine practice.

Not surprisingly, the benefits of Xpert testing were particularly evident among HIV-infected individuals in this study. The HIV epidemic has had a dramatic impact on TB case notifications in this region, and tackling HIV-associated TB will be key to controlling the TB epidemic [14]. HIV infection is associated with paucibacillary disease, and consequently, sputum smear microscopy performs particularly poorly in this patient group [15]. In Khayelitsha, many patients are diagnosed with TB on clinical grounds only. Indeed, given the prior lack of a TB diagnostic test with improved sensitivity in HIV-infected individuals, clinical TB diagnosis has been the focus of considerable training in the study clinic over many years prior to this study. Despite this, by 3 wk after presentation, close to double the proportion of HIV-infected participants had initiated TB treatment in the Xpert arm compared to the routine arm, and the proportion who started treatment without bacteriological confirmation was more than halved.

More extensive and more rapid treatment initiation might be expected to reduce mortality, particularly among HIV-infected patients with low CD4 cell counts. There was, however, no difference in mortality between study arms, overall or among HIV-infected individuals. The recent, larger XTEND study, also in South Africa, similarly found no difference in mortality with Xpert versus smear microscopy, although, contrary to the current study, Xpert did not significantly increase the proportion treated for TB [16]. Unfortunately, data on CD4 cell counts was inconsistently available, and we were not able to assess mortality in the subgroup of patients with low CD4 levels, where we might expect to see the greatest mortality benefit.

Xpert also resulted in limited clinical impact in the TB-NEAT study [8]. While initial default from treatment was reduced, very high (more than 40%) proportions of patients in both the Xpert and smear microscopy arms had received treatment by 8 wk, and there was no difference in TB-related morbidity in that large multicentre study. The authors postulate that any potential benefits from Xpert are masked by high levels of rapid empiric treatment initiation among smear-negative patients [17]. In our study population, empiric treatment is less common, and there is greater reliance on culture to subsequently confirm disease. While the provision of culture for all patients with presumptive TB might be considered ideal, the delay in receiving results means that most patients are treated empirically anyway. We may, however, have underestimated empiric treatment by including only participants producing sputum samples. Nonetheless, under these conditions, Xpert significantly reduced the proportion of patients who started TB treatment without bacteriological confirmation of disease, while simultaneously increasing the overall proportion starting treatment. This suggests that with Xpert more of the right patients initiated treatment.

While there is a high prevalence of microbiologically confirmed TB in our study population, the yield from sputum smear microscopy was particularly poor, primarily as most smear microscopy was conducted in the on-site laboratory. This is in contrast to previous routine data among the same population, where microscopy was performed in the central laboratory, and yield was higher [10]. Although this was a newly established laboratory, it passed all external quality assessment proficiency testing, and the laboratory technologist was experienced. This low microscopy yield reflects the reality of smaller, decentralised laboratories with a single laboratory technician [18]. Encouragingly, the quality of Xpert testing in this same laboratory was consistently high [4].

One of the major benefits of the Xpert MTB/RIF test is expected to be increased case detection for MDR-TB, through greater access to rifampicin susceptibility testing. No difference in confirmed rifampicin-resistant TB was found in this current study; however, the study was not powered to detect such a benefit, and significant efforts had been made to improve the detection of rifampicin resistance in this setting prior to this study [19]. One of the principal benefits of Xpert for patients with rifampicin-resistant TB will be more rapid treatment initiation, which might be expected to reduce both early mortality and transmission.

Recent reports have raised concern about false-positive Xpert results among patients who have been previously treated for TB [20]. Reassuringly, patients found to be Xpert-positive and culture-negative were not more common among those who were previously treated for TB. These results suggest that instances of false-positive results, although concerning, may be uncommon, and reinforce the need to interpret all diagnostic tests in the context of clinical presentation.

While every effort was made to ensure accuracy of follow-up data to confirm TB treatment initiation and deaths, it is possible that the data were incomplete, and some participants may have died or initiated treatment elsewhere without these events being captured in our data. However, this potential bias is not likely to be different across the study arms and is therefore not expected to influence the study conclusions. Another potential limitation is the lack of culture as the gold standard for TB diagnosis for all participants. However, the study aimed to assess the impact of Xpert in comparison with routine practice, and culture is generally available only for a proportion of those with presumptive TB, or not at all, in high-burden settings. The provision of culture for all participants would have, therefore, altered routine practice in this setting.

Controlling the TB epidemic in high-burden settings, particularly those with high HIV prevalence, such as in Khayelitsha, will require the implementation of new tools and programmes that do more than just provide incremental benefit over current strategies. While Xpert is considerably more costly than smear microscopy—with implementation expected to result in substantially increased health system costs in the short to medium term—it does offer the chance to impact TB morbidity and mortality over the long term [21]. This randomised trial demonstrates that implementation of Xpert for TB diagnosis in this setting has the potential to improve both programme and individual outcomes and therefore deliver the longer term population outcomes promised by modelling studies [22].

## Supporting Information

Datafile S1 **Minimum dataset used for analysis of data in this article.** All data have been anonymised.
(XLSX)

Text S1 **CONSORT checklist for reporting an abstract for a randomised trial.**
(DOC)

Text S2 **CONSORT checklist (2010 version) for reporting randomised trial data.**
(DOCX)

Text S3 **Original trial protocol.**
(DOC)

Text S4 **Supplementary trial protocol (original protocol appendix).**
(DOCX)

## Abbreviations

DST: drug susceptibility testing
ITT: intention to treat
MDR-TB: multidrug-resistant tuberculosis
TB: tuberculosis

## References

[1] World Health Organization (2013) Global tuberculosis report 2013. WHO/HTM/TB/2013.11. Geneva: World Health Organization.

[2] Lawn SD, Ayles H, Egwaga S, Williams B, Mukadi YD, et al (2011) Potential utility of empirical tuberculosis treatment for HIV-infected patients with advanced immunodeficiency in high TB-HIV burden settings. Int J Tuberc Lung Dis 15: 287–295. 21333094

[3] Helb D, Jones M, Story E, Boehme C, Wallace E, et al (2010) Rapid detection of Mycobacterium tuberculosis and rifampin resistance by use of on-demand, near-patient technology. J Clin Microbiol 48: 229–237. 19864480 10.1128/JCM.01463-09 PMC2812290

[4] Boehme CC, Nicol MP, Nabeta P, Michael JS, Gotuzzo E, et al (2011) Feasibility, diagnostic accuracy, and effectiveness of decentralised use of the Xpert MTB/RIF test for diagnosis of tuberculosis and multidrug resistance: a multicentre implementation study. Lancet 377: 1495–1505. 21507477 10.1016/S0140-6736(11)60438-8 PMC3085933

[5] World Health Organization (2010) Roadmap for rolling out Xpert MTB/RIF for rapid diagnosis of TB and MDR-TB. Geneva: World Health Organization.

[6] Steingart KR, Sohn H, Schiller I, Kloda LA, Boehme CC, et al (2013) Xpert(R) MTB/RIF assay for pulmonary tuberculosis and rifampicin resistance in adults. Cochrane Database Syst Rev 1: CD009593. 10.1002/14651858.CD009593.pub2 PMC4470352 23440842

[7] Kwak N, Choi SM, Lee J, Park YS, Lee CH, et al (2013) Diagnostic accuracy and turnaround time of the Xpert MTB/RIF assay in routine clinical practice. PLoS ONE 8: e77456. 24204834 10.1371/journal.pone.0077456 PMC3812224

[8] Theron G, Zijenah L, Chanda D, Clowes P, Rachow A, et al (2014) Feasibility, accuracy, and clinical effect of point-of-care Xpert MTB/RIF testing for tuberculosis in primary-care settings in Africa: a multicentre, randomised, controlled trial. Lancet 383: 424–435. 24176144 10.1016/S0140-6736(13)62073-5

[9] City of Cape Town Strategic Development Information and Geographic Information System Department (2013) City of Cape Town—2011 Census—Khayelitsha Health District. Cape Town: City of Cape Town.

[10] Médecins Sans Frontières (2011) Khayelitsha 2001–2011, activity report: 10 years of HIV/TB care at primary health care level. Geneva: Médecins Sans Frontières.

[11] Enarson D, Rieder HL, Arnadottir T, Trébucq A (2000) Management of tuberculosis: a guide for low income countries. Paris: International Union Against Tuberculosis and Lung Disease.

[12] Boulle A, Van Cutsem G, Hilderbrand K, Cragg C, Abrahams M, et al (2010) Seven-year experience of a primary care antiretroviral treatment programme in Khayelitsha, South Africa. AIDS 24: 563–572. 20057311 10.1097/QAD.0b013e328333bfb7

[13] World Health Organization (2010) Treatment of tuberculosis: guidelines (fourth edition). Geneva: World Health Organization.

[14] Lawn SD, Bekker LG, Middelkoop K, Myer L, Wood R (2006) Impact of HIV infection on the epidemiology of tuberculosis in a peri-urban community in South Africa: the need for age-specific interventions. Clin Infect Dis 42: 1040–1047. 16511773 10.1086/501018

[15] Getahun H, Harrington M, O'Brien R, Nunn P (2007) Diagnosis of smear-negative pulmonary tuberculosis in people with HIV infection or AIDS in resource-constrained settings: informing urgent policy changes. Lancet 369: 2042–2049. 17574096 10.1016/S0140-6736(07)60284-0

[16] Fielding K, McCarthy KM, Cox H, Erasmus L, Ginindza S, et al (2014) Xpert as the first-line TB test in South Africa: yields, initial loss to follow-up, proportion treated [abstract]. Conference on Retroviruses and Opportunistic Infections; 3–6 March 2014; Boston, Massachusetts, US. Top Antivir Med 22: 48–49.

[17] Theron G, Peter J, Dowdy D, Langley I, Squire SB, et al (2014) Do high rates of empirical treatment undermine the potential effect of new diagnostic tests for tuberculosis in high-burden settings? Lancet Infect Dis 14: 527–532. 24438820 10.1016/S1473-3099(13)70360-8

[18] Ridderhof JC, van Deun A, Kam KM, Narayanan PR, Aziz MA (2007) Roles of laboratories and laboratory systems in effective tuberculosis programmes. Bull World Health Organ 85: 354–359. 17639219 10.2471/06.039081 PMC2636656

[19] Cox H, Hughes J, Daniels J, Azevedo V, McDermid C, et al (2014) Community-based treatment of drug-resistant tuberculosis in Khayelitsha, South Africa. Int J Tuberc Lung Dis 18: 441–448. 24670700 10.5588/ijtld.13.0742

[20] Boyles TH, Hughes J, Cox V, Burton R, Meintjes G, et al (2014) False-positive Xpert((R)) MTB/RIF assays in previously treated patients: need for caution in interpreting results. Int J Tuberc Lung Dis 18: 876–878. 24902569 10.5588/ijtld.13.0853

[21] Menzies NA, Cohen T, Lin HH, Murray M, Salomon JA (2012) Population health impact and cost-effectiveness of tuberculosis diagnosis with Xpert MTB/RIF: a dynamic simulation and economic evaluation. PLoS Med 9: e1001347. 23185139 10.1371/journal.pmed.1001347 PMC3502465

[22] Langley I, Lin H, Egwaga S, Doulla B, Ku C, et al (2014) Assessment of the patient, health system, and population effects of Xpert MTB/RIF and alternative diagnostics for tuberculosis in Tanzania: an integrated modelling approach. Lancet Glob Health 2: e581–591. 25304634 10.1016/S2214-109X(14)70291-8

