# Supplementary material for: Impact of Xpert MTB/RIF for TB Diagnosis in a Primary Care Clinic with High TB and HIV Prevalence in South Africa: A Pragmatic Randomised Trial
Source: PLoS Med. 2014 Nov 25;11(11):e1001760. doi: 10.1371/journal.pmed.1001760 (PMC4244039; doi:10.1371/journal.pmed.1001760)
Supplement: Text S1 — CONSORT checklist for reporting an abstract for a randomised trial. (DOC) [file pmed.1001760.s002.doc]

**Items to include when reporting a randomized trial in a journal or conference abstract**

| **Item** | **Description** | **Reported on line number** |
| --- | --- | --- |
| Title | Identification of the study as randomized | 2 |
| Authors * | Contact details for the corresponding author |  |
| Trial design | Description of the trial design (e.g. parallel, cluster, non-inferiority) | 36 |
| Methods |  |  |
| Participants | Eligibility criteria for participants and the settings where the data were collected | 36-38 |
| Interventions | Interventions intended for each group | 36-38 |
| Objective | Specific objective or hypothesis | 38-39 |
| Outcome | Clearly defined primary outcome for this report | 38-39 |
| Randomization | How participants were allocated to interventions | 40-41 |
| Blinding (masking) | Whether or not participants, care givers, and those assessing the outcomes were blinded to group assignment | 40 |
| Results |  |  |
| Numbers randomized | Number of participants randomized to each group | 45-46 |
| Recruitment | Trial status | 45-47 |
| Numbers analysed | Number of participants analysed in each group | 45-47 |
| Outcome | For the primary outcome, a result for each group and the estimated effect size and its precision | 47-49 |
| Harms | Important adverse events or side effects |  |
| Conclusions | General interpretation of the results | 56-57 |
| Trial registration | Registration number and name of trial register | 43 |
| Funding | Source of funding | 58-59 |

**this item is specific to conference abstracts*
