# Supplementary material for: Impact of Xpert MTB/RIF for TB Diagnosis in a Primary Care Clinic with High TB and HIV Prevalence in South Africa: A Pragmatic Randomised Trial
Source: PLoS Med. 2014 Nov 25;11(11):e1001760. doi: 10.1371/journal.pmed.1001760 (PMC4244039; doi:10.1371/journal.pmed.1001760)
Supplement: Text S3 — Original trial protocol. (DOC) [file pmed.1001760.s004.doc]

| *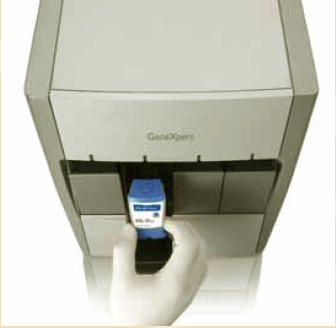* |  | **STUDY PROTOCOL** |
| --- | --- | --- |
|  |  |  |
|  |  | **Xpert^TM^ MTB/Rif Demonstration**  **Version 3; 6 August 2009** |
|  |  |  |
|  |  | Feasibility, impact and cost-efficiency of decentralizing molecular testing for detection of tuberculosis and rifampicin resistance using Xpert^TM^ MTB/Rif |

INVESTIGATORS

Andrew Whitelaw^1,2^ MBBCH, MSc, FCPath (Micro), Helen Cox^3^ BSc, MPH, PhD, Gilles van Cutsem^3^ MD MPH, Eric Goemaere^3^ MD PhD, Cheryl McDermid^3^ MD, Gerrit Coetzee^2^ MBBCh, FCPath (Micro), John Simpson^2^ MBBCh, FCPath (Micro), Catharina Boehme^4^M.D., PhD, Mark Nicol^1,2^ MBBCh, MMed, FCPath (Micro), PhD

^1^University of Cape Town, ^2^National Health Laboratory Service, ^3^Médecins Sans Frontières and ^4^Foundation for Innovative New Diagnostics

Contact Details:

Prof. Mark Nicol

Room 3.03.3 Wernher & Beit Pavilion

Institute of Infectious Diseases and Molecular Medicine

Faculty of Health Sciences

Anzio Road, Observatory, 7925

Tel +27 21 406 6083 Fax +27 21 406 6081

# PARTNER INSTITUTIONS

1. **South Africa (2 sites – Khayelitsha and Paarl)**

National Department of Health and National TB Programme

National Health Laboratory Services (NHLS)

University of Cape Town (UCT)

### Peru (2 sites)

National TB Programme (NTP) & Instituto Nacional de Salud (INS)

Instituto de Medicina Tropical “Alexander von Humboldt “, Universidad Peruana Cayetano Heredia (UPCH)

1. **Philippines (2 sites)**

National TB Programme (NTP)

Tropical Disease Foundation (TDF)

# ABSTRACT

The Xpert™ MTB assay is a rapid automated test (under 2 hours) for the detection of M. tuberculosis (MTB) and rifampicin resistance. The assay has undergone clinical validation in reference settings in South Africa, India, Peru, Germany and Azerbaijan. The assay was found to be highly sensitive and specific, detecting M. tuberculosis DNA in almost all smear-positive sputum specimens and 90% of smear-negative, culture-positive specimens. Thus the Xpert™ MTB appears to be suited for both rapid MDR screening as well as early case detection in smear negative TB patients. It is anticipated that the system will allow for on-the-spot diagnosis and TB treatment initiation based on one Xpert result. These findings need now to be confirmed in the intended setting of use.

STUDY DESIGN (3 phases)

1. Validation Phase (3 months enrolment): This validation phase aims to collect baseline data for the validation of Xpert™ MTB performance in this new environment. Test results not used for patient treatment.

2. Implementation Phase (3 months enrolment): Smear microscopy and Xpert™ MTB/Rif are used for TB treatment decisions. Culture will be performed by the reference laboratory for all cases.

3. Continuation Phase (6 months enrolment): Smear microscopy and Xpert™ MTB/Rif are used for TB treatment decisions. Selected conventional DST is done only for Xpert™ Rif resistant cases.

Patients included will be those with a clinical suspicion of pulmonary TB as well as those at risk of MDR-TB. Children <18 yrs and patients already on TB therapy will be excluded.

Sample collection and analysis

Sputum samples for investigation will be collected following standard practice at the clinic. Xpert™ MTB will be done on site (Khayelitsha) or at the local NHLS laboratory (Paarl). Samples from at least 150 patients will be evaluated per month, giving a total of at least 1800 patient samples. Patients will follow the normal flow within the clinic. Testing will be integrated into the routine activities of the clinic.

INTRODUCTION

Despite the enormous global burden of TB, approaches to TB diagnosis still rely on traditional methods that have major limitations. The disproportionate amount of smear-negative disease in sub-Saharan Africa, which shoulders two-thirds of the global burden of HIV/AIDS, has greatly complicated TB case detection and disease control. Multidrug-resistant (MDR) TB, defined as resistance to both isoniazid and rifampin, two of the most important “first-line” anti-TB drugs, is a rapidly growing problem, both in Eastern Europe and countries with high HIV prevalence. Even more alarming is the recently documented phenomenon of XDR (or extensively drug-resistant) TB, defined not only as resistance to isoniazid and rifampin but also to one second line injectable agent and a fluoroquinolone antibiotic. XDR TB is associated with significantly poorer treatment outcomes than MDR TB.

The low sensitivity of smear microscopy limits its impact on TB control. Culture methods are complex, slow and still only scarcely available in high endemic-countries. Drug susceptibility testing is even slower and more technically complex. While patients await diagnosis, their disease progresses with an increased chance of dying from tuberculosis and they continue to transmit drug-resistant TB to others, especially family members.

Early case detection is essential to interrupt transmission and to prevent the further spread of tuberculosis and multidrug-resistant tuberculosis. A new diagnostic approach is therefore urgently needed.

Only a small fraction of the estimated 489,000 MDR TB cases occurring each year have access to drug susceptibility testing DST. Key obstacles to DST expansion over the past years were 1) the complexity of available tools and the laboratory infrastructure required for their implementation; 2) the unaffordable price of better (but equally complex) technologies; and 3) the unavailability of rapid and simple tools for identification of resistance.

Although nucleic acid amplification tests (NAAT) for the rapid detection of TB and rifampicin resistance exist, their complexity, along with the labor demands, including the need for a high degree of technical support and well-trained staff, make them suitable for central reference laboratories only.

**DESCRIPTION OF Xpert™ MTB/Rif TEST**

The Xpert™ MTB/Rif assay performed on the GeneXpert™ MTB/Rif system is a rapid test for the detection of *M. tuberculosis* (MTB) and rifampicin resistance. The GeneXpert™ system consists of a GeneXpert instrument, personal computer and disposable fluidic cartridges. The system combines cartridge-based sample preparation with amplification and detection in a fully integrated and automated nucleic acid analysis instrument.

**CLINICAL PERFORMANCE OF Xpert™ MTB/Rif TEST**

After completion of development, the assay has undergone clinical evaluation in a statistically powered number of patients in reference settings in South Africa, India, Peru, Germany and Azerbaijan. As summarized in the table below, the Xpert™ MTB/Rif assay was found to be highly sensitive and specific, detecting *M. tuberculosis* DNA in almost all smear-positive sputum specimens and a very high percentage of smear-negative, culture-positive specimens. Rifampicin resistance and MDR was detected with high accuracy. Assay performance even remained good when analyzing a single Xpert™ MTB/Rif result in comparison to 4 culture results per patient. The performance was very homogenous across countries.

|  | Sensitivity  in S+C+ | Sensitivity  in S-C+ | Specificity  in Non-TB |
| --- | --- | --- | --- |
| Total | 99.5%  [98.4% - 99.8%] | 90.0%  [84.6% - 93.7%] | 97.9%  [96.4% - 98.8%] |

**Table: Per patient analysis, Xpert™ MTB/Rif sensitivity and specificity (3 Xpert, 3 smears, 4 cultures), preliminary study results.**

|  | Sensitivity in Rif resistant cases | Sensitivity in MDR cases | Specificity in Rif sensitive cases |
| --- | --- | --- | --- |
| Total | 96.1%  [92.4% - 98.0%] | 96.5%  [93.0% - 98.3%] | 98.6%  [97.2% - 99.3%] |

**Table: Per patient analysis, Xpert™ MTB/Rif sensitivity and specificity for Rif and MDR detection (3 Xpert, 3 smears, 4 cultures), sequencing data not taken into consideration; preliminary study results.**

In conclusion, Xpert™ MTB/Rif was not only suited for rapid MDR screening, but also seemed very useful for early case detection in smear negative TB patients. The high accuracy of a single Xpert™ MTB/Rif result per patient and the time to diagnosis of below 2 hours should allow for on-the-spot diagnosis and TB treatment initiation based on one Xpert™ MTB/Rif result only. These findings need now to be confirmed in the intended setting of use.

We will inform you as soon as the submitted manuscript with evaluation study results has been accepted for publication.

## MOTIVATION

In response to the growing problems of MDR and XDR TB, as well as the continuing epidemic of HIV-associated TB, WHO and its partners have called for greatly enhanced global capacity for mycobacterial culture and DST as well as for implementation of rapid methods for screening for MDR TB. This massive scale-up is thought to require that up to 2000 new culture-capable TB laboratories be established and 10,000 new laboratory workers be trained by 2015.

It is possible that much of this needed capacity could be addressed by molecular tests for case finding and MDR TB detection. With the WHO endorsement in 2008 of line probe assays (LPAs) for MDR TB screening, many countries are implementing this system. However, as noted above, the test is sufficiently complex that decentralized testing is not feasible. Therefore, the Xpert™ MTB/Rif assay is of great interest as it provides for highly accurate and simplified testing at a much lower level of the health system. Although it provides information only on rifampin resistance (in addition to detection of *M. tuberculosis* complex), in nearly all settings rifampin resistance is an accurate surrogate for MDR TB.

Although good performance of the Xpert™ MTB/Rif assay has been demonstrated in carefully controlled laboratory settings, data on testing in program conditions, as well as information about cost and patient/public health impact, are needed to inform public policy on the use of the system. These demonstration projects are intended to provide this information.

It is proposed that the Western Cape will be one of the demonstration sites for this multi-centre study. The partners will be the National Department of Health, the University of Cape Town, Médecins Sans Frontières, the National Health Laboratory Service (NHLS) and the Foundation for Innovative New Diagnostics (FIND).

## AIMS

The purpose of this demonstration project is to assess the implementation of Xpert™ MTB/Rif in decentralized laboratories in low- and moderate-income settings. The project aims at a) confirming the Xpert™ MTB/Rif performance in decentralized laboratories and b) demonstrating the feasibility, impact and cost-efficiency of using Xpert™ MTB/Rif at lower levels of the health system for enhanced detection of smear-negative TB and MDR TB. The data will be reported to national public health authorities and WHO in order to inform policy decision.

**Study hypothesis**

Fully automated molecular testing with integrated specimen processing by Xpert™ MTB/Rif is easy and robust enough to be decentralized and allows to significantly increase case detection rates for TB and MDR TB, as well as to shorten time to treatment initiation in comparison with the conventional diagnostic algorithm. Patient management based on a single Xpert™ MTB/Rif test, carried out while the patient is waiting for his/her result, decreases the drop out rate prior to diagnosis and the costs and barriers for the patient. The introduction of such testing may therefore be cost-effective for the health system when compared to conventional culture and DST costs.

**SPECIFIC OBJECTIVES**

1. **Clinical performance**
   - Measure increased yield in TB and MDR TB case detection rates compared to local routine diagnostic algorithm
   - Determine sensitivity and specificity compared to central culture and DST (preferable on liquid media).
2. **Operational performance**

- Assess robustness of Xpert™ reagents and equipment, maintenance and customer support needs, as well as minimal training needs
- Develop recommendations on how to integrate automated molecular testing in resource – poor countries using existing diagnostic algorithms for case detection and MDR TB screening.

1. **Cost-efficiency**

- Quantify “per test” and “per patient” costs for the health system compared to routine culture and DST

## METHODS

## Study design

The demonstration project will primarily assess the feasibility of using Xpert™ MTB/Rif in district laboratories.

## Study design

The demonstration project will primarily assess the feasibility of using Xpert™ MTB/Rif in district laboratories.

**Training & proficiency testing** (3-5 days):

During the on-site training phase, lab technicians from the district laboratory and the supervisor from the reference laboratory will receive training on the Xpert™ MTB/Rif assay from the FIND study coordinator. The competency if the staff to perform the assay will be assessed using stored, previously cultured samples from the evaluation study.

**Evaluation study (52 weeks)**

The study has been designed in a cluster randomized fashion, with each week considered as a separate cluster. Eligible TB suspects will be treated either on the basis of standard microscopy +/- culture and susceptibility testing (**Routine Arm**), or will be treated on the basis of the Xpert assay (**Xpert Arm**, with Xpert at times performed in conjunction with microscopy and culture as detailed below). The laboratory testing algorithm will alternate between Xpert and standard of care on a weekly basis, and the results sent back to the clinic will be acted upon irrespective of the test method. There are different phases to the study, as outlined in the table below.

**Phase 1:** 16 weeks

**Routine Arm (plus culture):**

2 sputum samples per patient.

- 1 sputum has standard smear microscopy at routine laboratory (either Paarl NHLS laboratory or on-site laboratory at Khayelitsha clinic).
- 1 sputum has microscopy and TB culture at reference laboratory (Greenpoint NHLS). Susceptibility will be done on clinical suspicion of MDR-TB

Treatment based on above results. Patients followed up at 2 and 6 months if they are smear or culture positive. Smear negative patients asked to return if symptoms persist. Smear negative, culture positive patients will be traced if possible.

**Xpert Arm (plus culture):**

2 sputum samples per patient

- 1 sputum for Xpert at routine laboratory
- 1 sputum for smear and culture at reference laboratory (DST performed if clinical suspicion of MDR-TB, or if Xpert test shows rifampicin resistance)

Treatment based on Xpert result, or culture result (in cases of Xpert negative, culture positive). Xpert or culture positive patients followed up at 2 and 6 months. Smear negative and Xpert negative patients asked to return if symptoms persist.

Phase 1 will allow us to evaluate the performance of the Xpert system in a clinic setting. The additional cultures performed will allow us to determine whether the performance of the assay in a routine setting is comparable to the performance described in a research setting. These are as described in Objectives 1b and 2a.

**Phase 2:** 16 weeks

**Routine Arm:**

2 sputum samples per patient

- 1 sputum for microscopy at routine laboratory
- 1 sputum for microscopy (routine laboratory), or microscopy plus culture +/- susceptibility testing (reference laboratory) at the clinic discretion (i.e., following standard practice)

Treatment based on microscopy and/or culture results, as per routine practice. Patients followed up at 2 and 6 months if they are smear or culture positive. Smear negative patients asked to return if symptoms persist. Smear negative, culture positive patients will be traced if possible.

**Xpert Arm:**

- 1 sputum for Xpert at routine laboratory
- 1 sputum for microscopy if Xpert positive, and for microscopy plus culture and DST if Xpert shows rifampicin resistance. The purpose of microscopy for the Xpert positive sputa is to provide a baseline microscopy result for follow-up purposes, as the national TB programme performs smear microscopy at follow up on smear positive cases. Xpert negative cases will have no culture performed.

Treatment based on Xpert results, Patients followed up at 2 and 6 months if they are Xpert positive. Xpert negative patients asked to return if symptoms persist.

Phase 2 is designed to assess the impact that introduction of the Xpert test will have on diagnosis and management of TB, as outlined in Objective 1a. Information from both this phase and phase 1 will be used to quantify the cost-effectiveness of the assay (Objective 3)

**Data collection**:

During phases 1 and 2, data will be collected at first presentation for all TB suspects, and data will be collected at the 2 and 6 month follow up visits for Xpert and/or smear and/or culture positive patients. The data in the CRF will be data that is normally collected as part of routine clinical practice. The purpose of the CRF is to allow us to record in a central database information that will allow us to assess the impact of the study in particular. This information thus includes how the diagnosis of TB was made, when TB was diagnosed, when a diagnosis of drug resistant TB was made, what additional investigations were performed, clinical response to therapy, HIV status when known.

**Phase 3:** 20 weeks

**No randomization**.

2 sputa per patient

- 1 sputum for Xpert at routine laboratory
- 1 sputum for microscopy if Xpert positive, and for microscopy plus culture and DST if Xpert shows rifampicin resistance. The purpose of microscopy for the Xpert positive sputa is to provide a baseline microscopy result for follow-up purposes, as the national TB programme performs smear microscopy at follow up on smear positive cases. Xpert negative cases will have no culture performed.

Treatment based on Xpert results, Patients followed up as per routine clinic practice (which may mirror the 2 and 6 month follow up described above, but case report forms will not be completed at these follow up visits)

Phase 3 will (in conjunction with phases 1 and 2) provide the best opportunity to develop recommendations on the integration of the Xpert assay into the routine diagnostic algorithm and will allow assessment of introduction of Xpert on clinic and laboratory workload.

Inclusion criteria

- Clinical suspicion of pulmonary TB (not on TB treatment)
- Re-treatment cases
- Relapses
- Non-converting PTB cases > 2-3 months
- Symptomatic contacts of known MDR cases

**Sample size**

Estimated number of Xpert™ MTB/Rif tests to be conducted over the course of the study:

Six demonstration project sites; 12 months: 10,800 – 14,400 Xp tests.

Four access project sites; 12 months: 7,200 - 9,600 Xp tests.

Overall, approximately ***20,000 sputum samples*** are expected to be tested by Xp during this project. Rationale:

It is expected that the average specimen load at participating district laboratories is 600 sputum samples per month. Approximately 400-450 of these samples will be from TB or MDR suspects, whereas 150-200 will be for monitoring of treatment follow up. The latter will not be eligible for this project. As per current diagnostic algorithm in the majority of countries, 2-3 sputa are submitted per TB suspect and 1 per MDR suspect. For the study, only one sputum sample will be tested by Xpert™ MTB/Rif per patient (when in an Xpert arm of the study). It is anticipated, based on data from the Khayelitsha clinic, as well as the Paarl clinics and Paarl laboratory, that each site sees between 45 and 50 TB suspects (new and previously treated cases) per week. For 32 weeks of the study, patients will receive an Xpert test on their sputum, resulting in 1600-1800 Xpert tests for the duration of the study.

**Study Sites**

The proposed study will be conducted at 1 clinic in Khayelitsha (Site B) and 3 clinics in Paarl (JJ du Preez, Klein Nederburg and Dalvale). These sites have been chosen for the following reasons:

- 1. The presence of an on-site or nearby NHLS peripheral laboratory. This study is specifically designed to assess whether the GeneXpert system will be appropriate and feasible for use in a peripheral laboratory or clinic setting. Site B is the only clinic with an on-site laboratory, making it ideal for this evaluation. Patients will be able to receive laboratory results on the same day. This is likely to make an enormous impact on the quality of patient care and result in significant reduction in the number of patients lost to follow-up. The three clinics in Paarl are less than 5km from the NHLS laboratory, so that results may, in some cases be available the same day, but at the very least on the following day.
  2. Patient numbers: we require a minimum of 150 TB suspects per month from each site.
  3. Proximity to Cape Town: this will allow staff from the relevant supervisory centres (NHLS and University of Cape Town) to visit regularly.

**Preparation and Participation of Health Services**

The proposed study will be cleared with the relevant clinic committees prior to commencing. The study nursing sister and medical officer will spend 2 weeks within the clinics prior to commencing to assess the best way of implementing the study without interfering with routine work, in consultation with the clinic workers.

The proposed study will be integrated into clinic activities so that it does not interfere with the routine activities of the clinics. We will aim to enhance clinic capacity in the following manner:

- 1. Patients will follow the normal flow within the clinic. Testing will be integrated into the routine activities of the clinic. We have received ethics approval for waiver of informed consent, since there is no risk and substantial benefit for the patients. There will therefore not be any additional time required for patient consent or information.
  2. We will provide one additional nursing sister or research worker per clinic as well as a clerical assistant / data capturer. These staff will assist with ensuring that records are complete, and that results are fed back to the relevant clinic staff in a timely manner. The only study-specific activities requiring additional health care worker time will be to ensure that the relevant data fields are captured. This will be performed by study staff. Study nursing staff will also contribute to the routine activities of the clinic, where the local clinic feels this to be appropriate.
  3. A study medical officer will be appointed. Part of his/her activities will include assisting with routine care of TB patients, where the local clinic feels this to be appropriate.
  4. The availability of GeneXpert results (with performance similar to that of TB culture) at the same clinic visit (or on the following day) will dramatically improve the ability to provide rapid and accurate diagnosis of tuberculosis at the clinics. Furthermore, identification of Rifampicin resistance will identify those patients at risk for MDR-TB. In all patients with a rifampicin resistant result, the test will be confirmed using Hain MTBDR*plus* testing for independent confirmation.
  5. The NHLS will be a partner in the proposed studies. Additional technicians/technologists (to be employed by the NHLS, but funded through this study) will be placed in both peripheral labs as well as in the Greenpoint lab to assist with the extra workload. It is anticipated that this additional staff will also contribute to routine work within these labs.

**Timelines**

| **Activity / Event** | **Date** |
| --- | --- |
| IRB approvals (phase 1 and 2) | By May 09 |
| Shipment of GeneXpert instruments & cartridges | May 09 |
| Validation Phase - enrolment | July 09 – Sep 09 |
| Implementation Phase – enrolment | Oct – Dec 09 |
| Continuation Phase | Jan 10 – Jun 10 |
| WHO expert committee meeting | Apr 10 (possibly Sep 10) |

## ETHICAL CONSIDERATIONS

During the various phases, patient management and TB treatment decisions will be based on either routine diagnostoic results, or the Xpert™ MTB/Rif results. For the Xpert results, the routine diagnostic algorithm will be modified. Ethical approval will therefore be obtained for all participating sites.

Benefits & Risks: Numerous published studies and a large-scale FIND demonstration project confirm the high accuracy of rpoB-based tests for rifampicin resistance detection. In June 2008, the Strategic Technical Advisory Group (STAG) of WHO has approved line-probe assays for MDR screening in developing countries. However, this use is restricted to central laboratories due to complexity and biosafety and equipment needs.

The Xpert™ MTB/Rif has the additional advantages of simplicity, reduced biosafety needs and much increased sensitivity which should allow using the assay for case detection in smear-negative patients as well as for MDR screening in decentralized settings. All results obtained so far have shown excellent assay performance.

Based on these results (as outlined in the Introduction), as well as on the fact the Xpert assay is currently licensed as a diagnostic test in Europe and has been recently introduced to the private sector in South Africa, we feel that the use of the Xpert assay to guide treatment decisions is justifiable and necessary, and that the use of the Xpert assay for a selection of patients provides at least as good a diagnostic test as the current standard algorithm (and probably a better diagnostic test). There is potential substantial benefit to participants in the GeneXpert arm (increased sensitivity in smear-negative cases) however, since this test is not as yet standard of care anywhere in the world it is considered ethical to randomize. There is no change to routine care in the control arm (except where culture is performed in addition during phase 1, which represents enhanced standard of care). The data generated from this study will be key to informing the cost/benefit analysis that we anticipate will motivate for the introduction of GeneXpert for the diagnosis of TB worldwide. This will represent a major advance in TB care and the single most important development in TB diagnostics in over 50 years.

Thus, participation in this project poses no foreseeable risk to patients. Furthermore, investigators will have no direct contact with patients. All data will be obtained from records collected by the clinical and laboratory staff and will be stripped of patient identifiers prior to entry into project databases. All study team members will be required to sign a confidentiality agreement that protects all patient information. No sample bank will be created. The stored pellets and culture isolates will only be needed to resolve discrepant cases. Finally, it must be ensured by all sites that patients diagnosed by Xpert™ MTB/Rif assay, including MDR TB cases, receive appropriate treatment according to program norms throughout the demonstration period.

Confidentiality: There are no risks with respect to confidentiality. Patient identifiers will be encrypted. Only authorized medical personnel at the study sites will have access to patient names.

## DISSEMINATION AND IMPLEMENTATION

Data from these projects will be shared with WHO Stop TB that is expected to issue guidance on the use of this assay in 2010. Following established procedures, WHO will convene an Xpert™ MTB/Rif committee to review available data on the use and performance of the Xpert™ MTB/Rif and draw up guidelines for the implementation of the assay in low and middle income countries. This guidance will be reviewed by the WHO Strategic and Technical Advisory Group (STAG) for TB in June 2010. Subsequent to STAG approval, WHO will issue formal guidance.

Further, data from this study will be used by the NHLS to guide possible implementation of this assay.

Results of research findings will be fed back to the relevant clinics, to the City and the Provincial Department of Health. Results will be published in peer reviewed journals and at international conferences.

## REQUIREMENTS FOR SPACE, EQUIPMENT AND STAFF FROM THE FACILITY

Since testing will follow the routine flow of work within the clinic, there will be no specific additional staff requirements. However, the study will provide staff as documented above to ensure that study records are completed and that specimen collection and flow is optimal. There will not be any specific additional space required for the research, apart from a willingness to accommodate the additional nursing sister within the area of the clinic dedicated to reviewing patients with suspected TB. The Xpert instrument will be placed within the relevant NHLS laboratories (Site B and Paarl).
