# Supplementary material for: Impact of Xpert MTB/RIF for TB Diagnosis in a Primary Care Clinic with High TB and HIV Prevalence in South Africa: A Pragmatic Randomised Trial
Source: PLoS Med. 2014 Nov 25;11(11):e1001760. doi: 10.1371/journal.pmed.1001760 (PMC4244039; doi:10.1371/journal.pmed.1001760)
Supplement: Text S4 — Supplementary trial protocol (original protocol appendix). (DOCX) [file pmed.1001760.s005.docx]

**Xpert^TM^ MTB/Rif Demonstration**

**Phase 2: Supplementary protocol**

August 2010

**Summary:**

GeneXpert MTB/Rif is a simple automated real-time PCR test for the diagnosis of TB and identification of rifampicin resistance, performed directly on sputum samples. In a recently completed evaluation study (phase 1) GeneXpert showed high sensitivity for both smear negative and positive disease. We aim to evaluate the impact of this diagnostic test on time to treatment and proportion of patients with undiagnosed TB, two months after initial presentation to the health care facility.

**Primary endpoint:**

Proportion of patients with untreated TB two months after enrolment (untreated TB defined as patients with TB diagnosed by smear or culture at the month follow up visit, but not before or patients in whom the diagnosis of TB had been confirmed (smear, culture, Xpert) previously but who had not yet commenced TB treatment at the 2 month follow up visit.

**Secondary endpoints:**

1. Time to appropriate TB treatment (defined as time between enrolment and commencement of appropriate first-line or drug-resistant TB treatment).
2. Mortality (all cause).
3. Time to diagnosis (defined as the time between enrolment and a confirmed diagnosis of TB based on smear, culture or Xpert).
4. Number of clinic visits prior to appropriate TB treatment.

**Experimental design and methods**

Study setting

The proposed study site is Ubuntu clinic (Site B) in Khayelitsha, Cape Town in the Western Cape province of South Africa. This clinic has been chosen in close collaboration with the local TB programme coordinator and consent for this study has been sought from the Provincial and City Health Authorities.

Study design

A cluster randomised controlled design (cluster unit = week of randomization) will be used for pragmatic reasons. Individual randomization would substantially interfere with clinic and laboratory workflow. In addition, this strategy will allow assessment of the impact of the proposed intervention on the clinic and laboratory workload.

Sample size calculation will determine how many of weeks of enrolment will be required. Each of these weeks will comprise a randomisation cluster. Weeks will be randomly allocated in equal numbers to the control arm or the GeneXpert arm, using a random numbers table.

Waiver of informed consent will be sought from the Research Ethics Committee. The sputum of TB-suspects (all new cases, relapse cases and treatment failures) who present to the study clinics during the intervention period will be sent to the laboratory. Sputum samples collected for purposes of monitoring treatment (smear or culture conversion) will be excluded.

Patient inclusion criteria

• Clinical suspicion of pulmonary TB (not on TB treatment)

• Re-treatment cases

• Relapse cases

• Symptomatic contacts of known MDR cases

Description of study arms:

*Weeks randomised to the GeneXpert arm:*

As per routine practice, 2 sputum samples per participant will be collected on different days or same day (according to standard clinic practice) and will be sent to the laboratory. The laboratory will perform the GeneXpert MTB/Rif test according to manufacturer’s specifications and provide the clinic with the result as per routine reporting procedures. If the GeneXpert result is negative, the second sputum sample will be discarded. If positive, smear will be performed on the second sample.

Culture (mycobacterial growth indicator tube [MGIT]) and drug susceptibility testing (DST, Hain Genotype MTBDRplus on positive cultures) will be performed only when specifically requested as part of routine clinical practice. On receipt of the result, the clinic staff will follow their routine practice in notifying the patient and initiating appropriate treatment. All rifampicin resistant results will be confirmed by culture and routine DST of an additional sputum sample. Patients with rifampicin resistant TB will be referred for initiation of therapy for drug-resistant TB.

*Weeks randomised to the control arm:*

Similarly, as per routine practice, 2 sputum samples per participant will be collected on different days or same day (according to standard clinic practice) and will be sent to the laboratory. The laboratory will perform fluorescent acid fast microscopy on sputum sediment and provide the clinic with the result per the routine reporting procedures. Culture (mycobacterial growth indicator tube [MGIT]) and DST (Hain Genotype MTBDR*plus* on positive cultures) will be performed only when specifically requested according to routine clinical practice (typically for cases with suspected drug resistance and for patients with negative smear but strong clinical suspicion of TB). On receipt of the result, the clinic staff will follow their routine practice in notifying the patient and initiating appropriate treatment.

At a minimum, the following data will be collected for all patients investigated for TB over the study period (at least 1 respiratory specimen sent for TB investigation):

- 1. The date when the TB-suspect first presented to the clinic with symptoms
  2. The date of each visit to the clinic by the TB-suspect, including visits for sputum collection only, up until and including the visit where therapy is initiated where relevant
  3. The date or dates on which a sputum sample is collected from a TB-suspect and sent to the laboratory for smear microscopy or TB culture, over the entire study period
  4. The date that results of smear or culture, reported to the clinic by the laboratory, are recorded in the TB register
  5. The date of initiation of appropriate therapy for TB where relevant
  6. The number of deaths and hospital admissions attributable to TB for patients who live in the community served by each clinic, as determined by regular perusal of the hospital admission lists and death registers in the region
  7. The date and time that sputum samples for TB-diagnosis from the randomized clinics are logged in at the laboratory, and the date and time that the final laboratory result report is generated

Using this data the following end-points will be determined:

- Number of clinic visits prior to appropriate TB treatment
- Time between patient presentation to clinic with symptoms and appropriate treatment of TB
- The number of TB cultures and smear microscopy requested per patient
- The laboratory TB-related workload as determined by the total time spent by the laboratory in processing samples for TB diagnosis

Patient follow-up visits

In addition, we will contact all patients with negative smear (control arm) or GeneXpert (intervention arm) results at 2 months after their initial clinic visit. Contact will first be attempted telephonically, failing which we will conduct a home visit at 2 months to determine the outcome of these patients. By means of an interview conducted by a study team member with either the participant or the participant’s caregiver in the case of a child, the outcome of each patient will be determined. In particular, the following data will be collected: TB treatment, presence of persistent symptoms, re-testing for TB, hospitalization or other morbidity. Symptomatic untreated patients will be referred to the local clinic for further investigation and testing (TB culture and GeneXpert).

Using this data, the following end-point will be determined:

- Number of undiagnosed cases of tuberculosis in the intervention and control arms
- Morbidity and mortality in patients discharged from clinics with negative TB investigations

The follow up is a key component of the study and will address the important issue of the subsequent outcome of patients with TB who initially test negative for TB. This group includes both patients with undiagnosed (smear-negative) TB as well as patients with an alternative diagnosis. There is no data on the outcome of these patients in South Africa at present. Apart from the ability to assess the impact of GeneXpert in reducing such mortality, this study will also provide important information for the health services on the outcome of patients who test negative for TB at their initial clinic visit.

Sample size

Sample size was calculated based on the original objective of increased yield in TB cases, given limited data available for which to estimate the primary endpoint. Clustering (by weeks) was accounted for by the introduction of an intra-cluster correlation coefficient of 0.05.

| Expected change in TB case yield among TB suspects | 20% + 10% |
| --- | --- |
| Significance | 0.05 |
| Power | 80% |
| Intra-cluster correlation coefficient | 0.05 |
| Design Effect | 3 |
| Required sample size per arm | 882 |

Assuming that 40 TB suspects are seen in the clinic per week, a total of 46 weeks would be required.

Data analysis

Given that this is a pragmatic trial under program conditions, aiming for minimal disruption to clinic functioning, analysis will be by ‘intention to treat’, with all patient assessed in the study arm based on the random allocation of weeks. The study endpoints will be compared across the arms by this method.

Chi-squared analysis will be used to compare proportions and time to event (survival) analysis methods will be used to assess hazards of events occurreing over time (eg. Appropriate treatment initiation).

Staff required

The clinical study team will consist of a medical officer/project manager, nurse coordinator and 4 clinical research workers.

Ethical considerations

Approval for this study will be sought from the UCT Ethics Committee.

Waiver of individual consent will be sought from the Ethics Committee on the following basis:

1. Since the GeneXpert test and the routine diagnostic algorithm have been shown to have at least comparable sensitivity to TB, no participant would be disadvantaged by being included in the study.
2. All medical management is provided by the routine health service and study activities do not represent an additional burden or risk to participants. There is no change to routine care in the control arm.
3. There is substantial benefit for all patients in the study. Patients in the GeneXpert arm will receive access to this novel diagnostic method with increased sensitivity in smear-negative cases. All patients will receive the benefit of clinical follow-up at two months. This will be of particular benefit for those patients with persistent symptoms, who will be further actively investigated for TB.
4. The data generated from this study will be key to informing the cost/benefit analysis that we anticipate will motivate for the introduction of GeneXpert for the diagnosis of TB worldwide. This will represent a major advance in TB care and potentially the single most important development in TB diagnostics in over 100 years.

The second ethical consideration is the question of whether it is ethical to randomize patients, since the GeneXpert test has been demonstrated to have superior sensitivity for smear-negative cases. We believe that this is ethical for the following reasons:

1. GeneXpert is not as yet standard of care anywhere in the world.
2. Strong data will be needed to persuade policy makers of the potential benefit of GeneXpert testing for all patients with suspected TB. This test will be considerably more expensive than smear microscopy (even though preferential pricing for high burden countries has been negotiated). As a result, there is need for unambiguous data with hard end-points to motivate for this change in policy. Simply demonstrating improved analytical performance will not persuade policy makers.

Individual consent will be obtained from participants for the two month follow-up at the follow-up visit or interview. Due to the large number of collective individual patients expected in the study clinics and logistic constraints on staff at these busy clinics, consent of this group of participants is not possible at an earlier stage. A potential ethical concern here is that some identifiable information -such as name, address and sputum results- are gathered by the study team prior to consent being given by the participant. This information is required for operational needs.

To minimize this potential breach of confidentiality, the following measures will be taken:

- no analysis or data-entry will be done on any information obtained prior to individual informed consent;
- if a patient does not give consent for this part of the study, the identifiable information obtained for this patient will be destroyed and not analyzed;
- all study team members will be required to sign a confidentiality agreement that protects all patient information.

Furthermore, as already discussed, the follow-up visit will provide valuable information for the health services and will provide data on a critical outcome measure of this study (morbidity and mortality ascribed to undiagnosed TB). We anticipate that this study will have a significant impact on the effectiveness of diagnosing TB and lead to considerable benefit for the population as a whole and to individual TB patients. Therefore the study holds a favourable risk-benefit ratio for the study population. Moreover, there is substantial benefit for patients with persistent symptoms, who will be identified at the follow-up visit and reinvestigated for TB.

Verbal informed consent will be requested in the case of telephonic interviews, whilst written informed consent will be taken during home or clinic visits. If need be, the study team member will return after 24 hours to give the participant time to consider their decision.

Expected outputs

Results from this study are expected to inform the National Health Laboratory Service (NHLS) on further implementation of GeneXpert. Results of research findings will be fed back to the clinic, and the City of Cape Town and Provincial Departments of Health. Results will be published in peer-reviewed journals and at international conferences.
